# Physical and Linkage Maps for *Drosophila serrata*, a Model Species for Studies of Clinal Adaptation and Sexual Selection

**DOI:** 10.1534/g3.111.001354

**Published:** 2012-02-01

**Authors:** Ann J. Stocker, Bosco B. Rusuwa, Mark J. Blacket, Francesca D. Frentiu, Mitchell Sullivan, Bradley R. Foley, Scott Beatson, Ary A. Hoffmann, Stephen F. Chenoweth

**Affiliations:** * Department of Genetics & Department of Zoology, University of Melbourne Victoria, Australia; † School of Biological Sciences, The University of Queensland, QLD 4072, Australia; ‡ Department of Primary Industries (Victoria), Knoxfield, Victoria, 3180 Australia; § School of Biological Sciences, Monash University, Victoria 3800, Australia; ** School of Molecular and Microbial Sciences, The University of Queensland, 4072, Australia; †† Department of Molecular and Computational Biology, University of Southern California, Los Angeles, CA 90089

**Keywords:** linkage map, montium, RNA-Seq

## Abstract

*Drosophila serrata* is a member of the *montium* group, which contains more than 98 species and until recently was considered a subgroup within the *melanogaster* group. This *Drosophila* species is an emerging model system for evolutionary quantitative genetics and has been used in studies of species borders, clinal variation and sexual selection. Despite the importance of *D. serrata* as a model for evolutionary research, our poor understanding of its genome remains a significant limitation. Here, we provide a first-generation gene-based linkage map and a physical map for this species. Consistent with previous studies of other drosophilids we observed strong conservation of genes within chromosome arms homologous with *D. melanogaster* but major differences in within-arm synteny. These resources will be a useful complement to ongoing genome sequencing efforts and QTL mapping studies in this species.

Species of the genus *Drosophila* have a long history as experimental organisms in genetics research (Bridges 1916; Painter 1934) because of their worldwide distribution, easy maintenance in the laboratory, short generation times, and polytene chromosomes. Polytene chromosome maps constructed initially for *Drosophila melanogaster* (Bridges 1935; Lefevre 1976) and later for numerous other *Drosophila* species allowed the physical placement of genes in many types of genetic studies and provided the basis for modern genomics (Schaeffer *et al.* 2008). *D. melanogaster* has been developed as a standard species for genomics research, and its genome has now been extensively mapped and sequenced (Adams *et al.* 2000), as have the genomes of an additional eleven *Drosophila* species (Clark *et al.* 2007). Despite the continued development of genomic resources for the genus, the *montium* group has not yet been considered genomically. This group contains an estimated 98 species (Brake and Bachli 2008) and has long been considered a subgroup within the melanogaster group (Lemeunier *et al*. 1986). This relationship is now being reevaluated and it has been proposed to raise its status to that of species group (Da Lage *et al.* 2007). Its ancestors are thought to have separated from the group giving origin to *D. melanogaster* about 40MY ago (Tamura *et al.* 2004). It has been suggested that *montium* species have retained the ancestral genomic organization compared with *D. melanogaster* (Scouras 1996).

One member of the *montium* group, *D. serrata*, has been particularly well studied from an evolutionary standpoint. *D. serrata* has a broad geographical distribution, ranging from Papua New Guinea to south eastern Australia, and has emerged as a powerful model for addressing evolutionary questions such as the evolution of species borders (Blows and Hoffmann 1993; Hallas *et al.* 2002; Magiafoglou *et al.* 2002; Van Heerwaarden *et al.* 2009) and adaptation to climatic variables (Frentiu and Chenoweth 2010; Kellermann *et al.* 2009). The species has also been used to investigate the consequences of sexual selection (Hine *et al.* 2002), the evolution of mate recognition (Higgie *et al.* 2000), the evolution of sexual dimorphism (Chenoweth *et al.* 2008), sexual conflict (Delcourt *et al.* 2009), and the evolution of male mate choice (Chenoweth and Blows 2003; Chenoweth *et al.* 2007). Its multiple cuticular hydrocarbons, which serve as contact pheromones, have been extensively studied in the development of multivariate quantitative genetic approaches for exploring genetic constraints on adaptation (Blows *et al.* 2004; Chenoweth *et al.* 2010; McGuigan *et al.* 2011).

Despite the importance of this species as a model for evolutionary research, our poor understanding of its genome remains a significant limitation. Although a polytene chromosome map has been available for over two decades (Mavragani-Tsipidou and Scouras 1990), physical mapping has been confined to a small number of genes. Most of the genes are distributed on two chromosome arms with comparisons being made with other closely related species (Drosopoulou *et al.* 1996, 1997, 2002; Drosopoulou and Scouras 1995, 1998; Pardali *et al.* 1996; Scouras 1996). Inversion polymorphisms have been studied in *D. serrata* (Stocker *et al.* 2004) and the closely related *D. birchii* (Baimai 1970) and an expressed sequence tag (EST) library has recently been developed (Frentiu *et al.* 2009). Here, we provide a greatly expanded physical map, constructed by using *in situ* hybridizations to *D. serrata* polytene chromosomes of gene regions from *D. melanogaster*, *D. serrata* EST clones and polymerase chain reaction (PCR)-amplified *D. serrata* sequences. In addition we provide a first-generation linkage map based on EST-derived SNPs that will guide future quantitative trait locus (QTL) mapping studies and genome sequencing efforts in this emerging evolutionary model species.

## Materials and Methods

### Physical map construction

Laboratory lines of *D. melanogaster* and *D. serrata* were maintained at 19° in a constant light incubator. Several *D. melanogaster* lines were used. One was a chromosomal inversion-free long-term laboratory line, *D20*, whereas other lines had some of the common inversions found in this species, which helped in arm identification. The standard *D. melanogaster* cytogenetic map published on Flybase was used to localize the *D. melanogaster* probe sequences. The *D. serrata* line, *W31*, was collected from Wollongong, in eastern Australia, and determined to be inversion-free based on cytological comparisons (Stocker *et al.* 2004). Its polytene chromosome banding sequence was identical to the map published by Mavragani-Tsipidou and Scouras (1990) and used in the current study for allocating physical markers. However, for ease in this and future comparative mapping studies, we have changed the chromosome arm numbering from that of the original *D. serrata* map to better correspond to the *D. melanogaster* map. In the original map, the 2L and 3R arms of *D. serrata* were switched with respect to gene positions in *D. melanogaster* (Mavragani-Tsipidou and Scouras 1990). This was initially noted in the study by Drosopoulou *et al.* (1996). We have, therefore, exchanged the names of those two arms and changed the sequence of region numbers so that they are in better agreement with the *D. melanogaster* map.

Slides of *D. melanogaster* and *D. serrata* salivary gland chromosomes were prepared according to Ashburner (1989) and stored in 100% ethanol at −20°. They were removed from the ethanol, dried, and checked for chromosome spreads just before use. We used two different techniques for probe labeling. The most frequently used was nick translation. The plasmid and insert as well as some of the PCR-amplified probes were labeled according to the Invitrogen BioNick labeling system. These PCR-generated probes were amplified with primers designed from *D. serrata* ESTs. Some probes were also labeled by DOP PCR using the Roche Dig High Prime Biosynthesis Kit and primers designed for *D. melanogaster* intron/exon regions that had generated sequences in molecular studies on *D. serrata* genomic DNA.

The first amplification was a normal PCR using the specific primers and *D. serrata* genomic DNA. The amplified band of the expected size was excised from the gel and the DNA eluted. A second PCR was then performed on the amplified product with Dig-11-dUTP substituted for part of the dTTP and the specific primers. An aliquot of the product was run on a polyacrylamide gel together with the non-Dig−substituted PCR product to check for the presence and size shift of the Dig substituted sequence. The Dig substituted product was cleaned by spin column and used in the *in situ* technique.

In cases in which several bands were observed on the polyacrylamide gel after the Dig-substituted amplification, the band of the expected size was excised from the gel and the DNA extracted by mashing the gel slice in diffusion buffer (0.5M ammonium acetate, 10mM magnesium acetate, 1mM EDTA, pH 8.0, 0.1% sodium dodecyl sulfate), soaking it at 50° for 30 min in this buffer, vortexing briefly, and centrifuging the supernatant through a shredder column (QIAGEN) to remove gel fragments. This supernatant was subsequently used in the *in situ* hybridizations.

The *in situ* hybridization technique has been described previously (Stocker *et al.* 1993, 2006). Probe solution contained 40% or 50% formamide, 2× SSPE, and 0.05 µg/µl salmon sperm DNA. Five percent dextran sulfate or polyethylene glycol was often added to facilitate hybridization. Chromosome preparations were steam denatured together with the probe at 75° for 10 min followed by overnight incubation in a humid chamber at either 37° or 32° (for some heterologous probes). Posthybridization washes were as follows: 2 times, 5 min each in 2× SSC at room temperature; 5 min in 2× SSC at 42°; 5 min in 50% formamide/2× SSC at 42°; 2 times, 5 min each in 2× SSC at room temperature and then storage in 1× TBS overnight at 4°. Blocking and antibody reactions were subsequently carried out as in Stocker *et al.* (1993, 2006), either using Superblock, goat α-biotin and rabbit anti-goat rhodamine (Pierce) for the nick-translated probes, or a Fluorescent Antibody Enhancer Set for Dig Detection containing blocking solution, mouse α-Dig, anti-mouse Ig Dig, and anti-Dig fluorescein (Roche). Chromosome preparations were mounted in Vectashield (Vector Laboratories) containing 1.5 µg/ml DAPI (Roche) and examined with a Zeiss Photomicroscope using the Axiovision analysis program.

The locations of all *D. melanogaster* probes were checked on *D. melanogaster* chromosomes. Many of the probe sequences were in pBluescript. *Dca* and *white* were in a pUAST vector. The position of *Dca* was identified by comparing signals obtained with and without the *Dca* insert. Only one signal was obtained on *D. serrata* chromosomes with this vector minus *Dca*. This signal was located on the X-chromosome, and its strength as well as its position near a heterochromatic band suggested that it was identifying the *white* gene that is a large component of this vector. *ATP7* full length was in the Pac vector, *RpLP2* in pBR 332, *eve* in pGem, and *engrailed* in λgt10. pAGEN1 plasmids containing *D. serrata* sequences were also labeled by nick translation and came from an EST library (Frentiu *et al.* 2009). They were inserted in pAGEN1. These sequences were also used in the linkage map. Some of the *D. serrata* EST probes were hybridized to *D. melanogaster* chromosomes and gave signals in the anticipated region.

Sequences labeled by DOP PCR were CG14616 (*lethal(1) Go196*), CG4717 (*knirps*), CG9734 (*globin1*), CG8740, CG17559 (*donut*), and CG2165 (*C07*) whereas all remaining sequences were labeled using nick translation. Some sequences were labeled by both methods and were verified to hybridize to the same chromosome site. CG8740 gave a strong hybridization signal at a site on the *D. melanogaster*-homologous arm of *D. serrata*. However, another hybridization site for this probe was observed within the chromocenter. It would appear that some similar sequences are located in chromocentric heterochromatin.

### Linkage map construction

#### Crossing and genotyping:

We performed a reciprocal F2 intercross between two highly inbred lines of *D. serrata* derived from two natural populations spanning extremes of the species’ eastern Australian distribution (Cooktown: *CTN42*, Forster: *FORS4*). Because a large number of chromosomal inversions have been reported in *D. serrata* (Stocker *et al.* 2004), we verified that the lines were homosequential by using polytene chromosome squashes before crossing using the protocols described previously. For SNP discovery, we used Illumina RNA-sequation (35-bp reads) on cDNA created from RNA extracted from three-day-old adult flies from each of the two lines (*CTN42*: 14.9 million reads, *FORS4*: 3.8 million reads). We assembled ESTs from the two lines separately using Edena 2.0 (Hernandez *et al.* 2008) in “strict” mode using a minimum overlap value of 21 bp resulting in 23,081 ESTs from *CTN42* (N50 = 322 bp) and 922 from *FORS4* (N50 = 276 bp). Initial SNP discovery was achieved by then aligning the ESTs to each other using MUMmer version 3.2 (Kurtz *et al.* 2004). We then designed oligos for a subset of 65 SNPs and validated them via bidirectional Sanger sequencing of PCR amplicons from *CTN42* and *FORS4* lines. We annotated the ESTs using BLAST against the genome of *D. melanogaster* following the procedure in Frentiu *et al.* (2009).

SNP genotyping was performed using the SEQUENOM MassARRAY platform. Two multiplexes were used for genotyping 61 SNPS. For each multiplex assay approximately 10 ng of genomic DNA was used. DNA was extracted from whole fly bodies using a standard phenol-chloroform method. We removed three invariant SNPs from the 61 markers: s10, s36, and s37. We did not detect any difference in genotype calls between three replicates of each parental DNA sample, which is consistent with a genotyping error below 1% for these assays.

#### Analyses:

Markers were assigned to linkage groups using Joinmap 4.0 (Van Ooijen 2006) beginning at LOD = 4.0 and ending at 10.0. This approach assigned 58 markers to four linkage groups encompassing the X and the second, third, and fourth (dot) chromosomes. Map construction was performed using a least squares approach implemented in the regression option in Joinmap. We used a Kosambi (1943) mapping function. All other parameters were retained as default. Linkage maps were plotted with MapChart (Voorrips 2002).

We tested for transmission ratio distortion (TRD) using χ^2^ tests with a significance threshold of α = 0.05. Bonferroni corrections were not applied as physical linkage between markers on the same chromosome violates the assumption of independence between tests. We did not exclude markers showing TRD on the basis of χ^2^ test results for three reasons. First, many of the typed autosomal SNPs showed distortion from Mendelian expectations 1:2:1 at the nominal significance level. Second, we were unable to map large portions of the genome when the α = 0.05 criterion was used, resulting in unusually short maps. Third, one of our ultimate goals was to perform QTL mapping on the F2 cross. QTL analysis is not necessarily negatively affected by distortion (Xu 2008) and distortion can, in some circumstances, increase power. The Joinmap 4.0 program uses as default, a method for assessing linkage (“Independence LOD”) that is not sensitive to TRD. We therefore followed an approach similar to Muchero *et al.* (2009) that involved setting a minor allele frequency cut-off criterion for each SNP as a basis for marker exclusion. We used a minor allele frequency cut-off of 0.25.

## Results and Discussion

### Physical map

**Figure 1:**
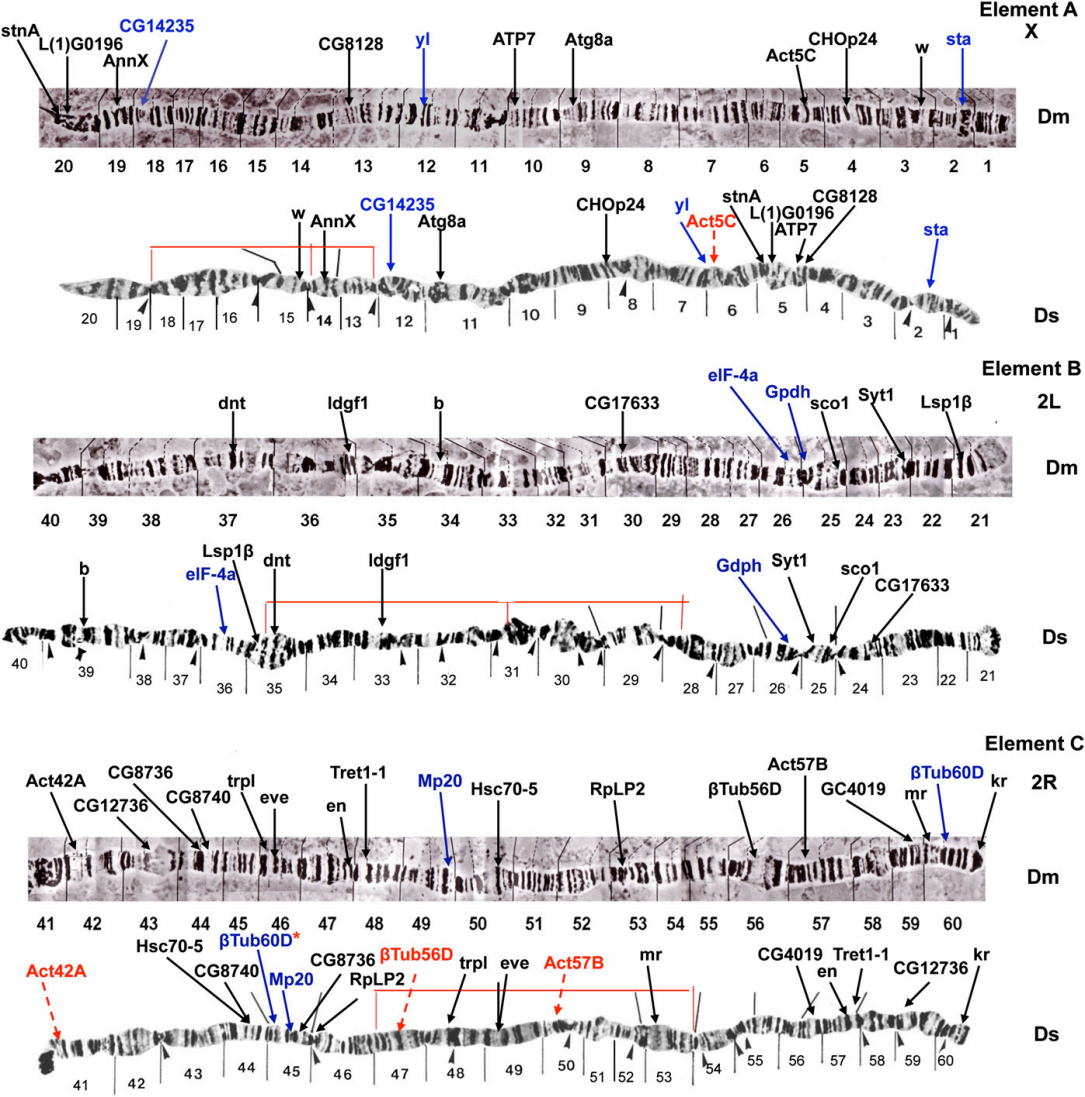
*D. serrata* and *D. melanogaster* polytene chromosomes showing mapped location of genes via *in situ* hybridization arranged in order of Mueller elements (Elements A–C in Figure 1 and Elements D–F in Figure 2). The *D. melanogaster* map is from Lefevre (1976), and the *D. serrata* map has been adapted from Mavragani-Tsipidou and Scouras (1990). Gene locations are indicated by arrows. Genes in red were mapped by Drosopoulou and Scouras (1995), Drosopoulou *et al.* (1996, 1997, 2002), Pardali *et al.* (1996), Drosopoulou and Scouras (1995, 1998) and are included for completeness. These genes were originally mapped to regions only. We have sometimes been able to give them a more defined position through examination of the original photographs. Genes in blue are SNP sequences also included on the linkage map. One of these genes, *βTub60D*, was also mapped by Drosopoulou *et al.* (2002). Thin red lines indicate inversions found along the eastern Australia *D. serrata* cline (Stocker *et al.* 2004). Black lines over *D. serrata* chromosomes and arrowheads beneath the chromosomes were designated as inverted repeat regions and weak points respectively by Mavragani-Tsipidou and Scouras (1990).

**Figure 2:**
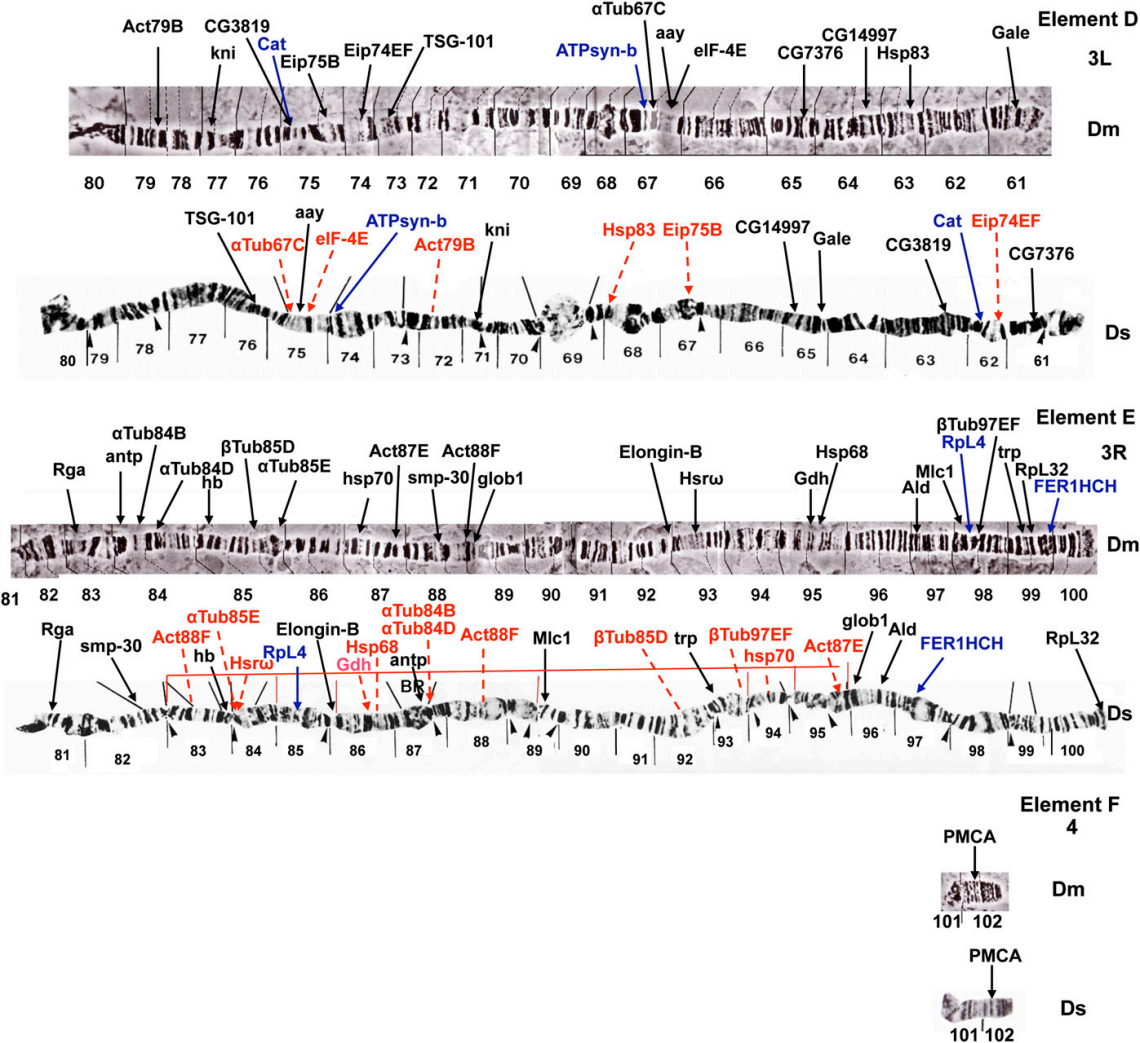
* D. serrata* and *D. melanogaster* polytene chromosomes showing mapped location of genes via *in situ* hybridization arranged in order of Mueller elements (Elements D–F). The *D. melanogaster* map is from Lefevre (1976), and the *D. serrata* map has been adapted from Mavragani-Tsipidou and Scouras (1990). Gene locations are indicated by arrows. Genes in red were mapped by Drosopoulou and Scouras (1995), Drosopoulou *et al.* (1996, 1997, 2002), Pardali *et al.* (1996), Drosopoulou and Scouras (1995, 1998) and are included for completeness. These genes were originally mapped to regions only. We have sometimes been able to give them a more defined position through examination of the original photographs. Genes in blue are SNP sequences also included on the linkage map. Thin red lines indicate inversions found along the eastern Australia *D. serrata* cline (Stocker *et al.* 2004). Black lines over *D. serrata* chromosomes and arrowheads beneath the chromosomes were designated as inverted repeat regions and weak points respectively by Mavragani-Tsipidou and Scouras (1990).

**Figure 3:**
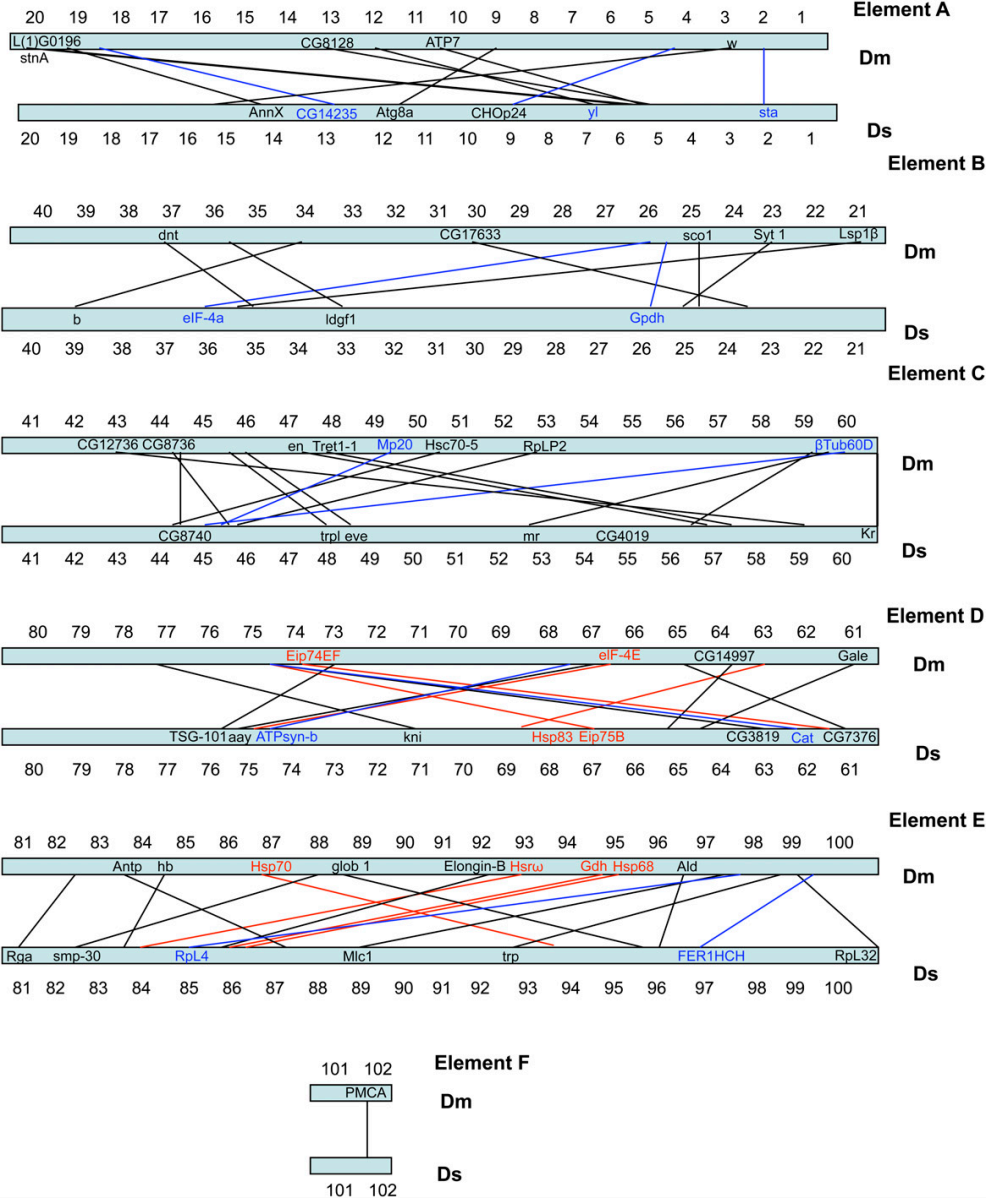
Schematic drawing showing conservation of synteny with shuffling of genes between *D. serrata* and *D. melanogaster* physical maps for the five chromosome arms. The numerous actin and tubulin genes mapped by Drosopoulou and Scouras (1995), Drosopoulou *et al.* (1997, 2002) are not included in this drawing.

In terms of large-scale genome architecture, arm number and chromosome shape have been maintained between *D. serrata* and *D. melanogaster*. When combined with previous efforts, our physical map places 78 genes on the *D. serrata* genome (Drosopoulou *et al.* 1996, 1997, 2002; Drosopoulou and Scouras 1995, 1998; Pardali *et al.* 1996), (Mueller element A (X): 12, Mueller element B (2L): 9, Mueller element C (2R):17, Mueller element D (3L): 15, Mueller element E (3R): 24, Mueller element F(4):1, Table S1). When *D. serrata* and *D. melanogaster* are compared, almost complete chromosomal arm synteny is observed for the genes mapped (Figure 1, Figure 2, and Figure 3). Such arm level synteny is common, even in distantly related *Drosophila* species, because uncommon pericentric inversions and translocations cause duplications and deletions in gametes, but more common paracentric inversions usually do not (Krimbas and Powell 1992; Ranz *et al.* 2001). The *actin* gene family showed one additional signal on element E in *D. serrata* that was not present in *D. melanogaster* (Drosopoulou *et al.* 1997) (Act88F; Figure 2). This could be attributable to the use of a heterologous probe in mapping this gene family. Differences in signals for heterologous and homologous probes were observed for the *β-tubulin* genes in *montium* species (Drosopoulou *et al.* 2002; Drosopoulou and Scouras 1995; Scouras *et al.* 1992).

Although chromosomal arm synteny has been conserved, there has been substantial positional gene change between *D. serrata* and *D. melanogaster* during the 40MY that have separated them (Figure 3). Extensive shuffling is also apparent when the positions of genes mapped in different members of the *montium* group are compared (Drosopoulou *et al.* 1997; Drosopoulou and Scouras 1995). This shuffling is most likely attributable to the occurrence of numerous large and small paracentric inversions. Some of these inversions could be caused by movements of transposable elements, which have been observed to be involved in restructuring events in chromosomes of *Drosophila* and other organisms (Gray 2000; Lim and Simmons 1994). Most transposable element–induced chromosomal rearrangements reported in *Drosophila* have been within-arm changes, possibly because of conformational restrictions of the chromatin (Lim and Simmons 1994) or lack of viability of the products of inter-arm arrangements (Gray 2000).

Although many of the genes studied now occur at very different positions on their chromosome arm, some groupings are still maintained in *D. melanogaster* and *D. serrata* (Figures 1 and 2). On the X-chromosome, *stnA* and *L(1)G0196* appear to have changed position but are still located next to each other in both species. This is also true for *Syt*, *sco1*, and *gdph* of arm 2L, *Tret1-1* and *en*, and *trpl* and *eve* of 2R; *ATPsyn-b*, *αTub67C*, *aay*, and *eIF-4E* of 3L; and *Hsp68* and *Gdh* of 3R. *Hsp68* and *Gdh* have maintained a close relationship in all but one of the six montium species compared by Drosopoulou *et al.* (1997). These two genes are located near an inversion breakpoint in both *D. melanogaster* and *D. serrata*, and this may be one factor keeping them together.

Duplication/deletion events may also have played a role in the chromosomal changes that have occurred between the two species, and transposable elements have been implicated in some of these (Gray 2000; Lim and Simmons, 1994). For example, *trpl* and *eve* on element C (Figure 1) appear to be located closer together in *D. melanogaster* than in *D. serrata*. The region separating these two genes in *D. serrata* has the appearance of a repeated region. It would be interesting to know the position of these two genes in other members of the *montium* group.

Certain genes that we examined localize to the same chromosomal positions in *D. melanogaster* and *D. serrata*. On the X-chromosome, *sta* is located in region 2 of both species, and the region itself has a similar appearance (Figure 1). On the 2R arm, *kr* is located near the nonchromocentric telomere in both species (Figure 1). This telomeric location suggested that genes at the ends of chromosomes might not be easily shuffled by paracentric inversions. However, *RpL32* is located near the end of the *D. serrata* 3R arm but has a more internal location in *D. melanogaster* (Figure 2). Despite the gene movements that have occurred between *D. melanogaster* and *D. serrata*, the polytene chromosome structure of short regions around some homologous genes and noncentromeric telomeres often has a similar appearance. Examples are short regions around *AnnX*, *yl*, and *CHOp24* on the X-chromosome; regions around *dnt*, *ldgf1*, *b*, and *CG17633* on arm 2L; regions around *Act57B*, *Tret1-1*, *en*, and *Act42A* on arm 2R; regions around *Act79B* and *kni* and *aay*, *eIF-4E*, *αTub67C*, and *ATPsyn-b* on chromosome 3L; and regions around *Rga*, *Ald*, and *Gdh* and *Hsp68* on arm 3R (Figures 1 and 2). However, no extensive homosequential regions are apparent in these two species.

#### Broad-scale synteny with other Drosophila species:

Although our primary goal was to compare *D. serrata* and *D. melanogaster*, the availability of a further 11 sequenced *Drosophila* genomes enabled us to also compare syntenic relationships, at least in terms of within-arm order, in a wider phylogenetic context. Using Flybase, we compared the order of genes on the *D. serrata* physical map to the order of their orthologs in the 12 available genomes (Schaeffer *et al.* 2008) (Table S3). Our results were largely consistent with those obtained by Bhutkar *et al.* (2008) with the exception that we did not observe greater positional rearrangements in the *Sophophoran* subgenera than in the *Drosophila* subgenera, likely because of the smaller number of genes analyzed. Arm-level synteny has been conserved for almost all genes included on the *D. serrata* physical map, except where identifiable chromosome structural changes have occurred in other species. For example, the pericentric and paracentric inversions in two members of the melanogaster species group, *D. erecta* and *D. yakuba* have mixed genes from the B and C elements.

When *D. serrata* is compared with the species in the *melanogaster* group, gene order is poorly conserved for almost all regions of the genome. Despite this poor conservation, some similarities are apparent. For example, the similarity at the telomeric end of element A extends through the *melanogaster* group to *D. serrata*. The change caused by inversions in this region can also be observed in *D. yakuba*. There appear to be more changes caused by paracentric inversions between *D. serrata* and the *melanogaster* group species; however, they cannot be followed by banding similarities. From an examination of gene order, it appears that there has been an inversion between CG12500 and the centromere, with another possible break above CG32672, which has exchanged the position of genes in the two regions of the A element (Table S3). A subsequent inversion could be proposed between CG14792 and CG2759 to bring CG2759 nearer the centromere. However, numerous other inversions in these regions must have occurred to explain the shuffling of most other genes in this element.

In the C element, *D. serrata* shows greater similarity with *D. ananassae* than with the *melanogaster* group species. However, the inversions that have positioned the genes in this arm have obviously been different in the lines leading to the two species. For element E, the most densely mapped chromosome, a small group of genes are syntenic in the melanogaster species group and *D. serrata*. This group, in the order CG1028, CG1913, CG2512 telomere to centromere, in *D. melanogaster*, *D. simulans*, *D. sechelia*, and *D. erecta* has been maintained in *D. serrata*, but the genes reside in different regions. In *D. yakuba*, pericentric inversions appear to have reversed the order to CG2512, CG1913, CG1028 (Table S3). In *D. ananassae* and species more distantly related to *D. melanogaster*, this group of genes has been completely broken up. Although our comparison of syntentic relationships in this study was limited to relative gene order, future sequencing of the *serrata* genome should give a much more accurate picture of the gene changes that have occurred and their relationship to gene positions across the *Drosophila* phylogeny.

### Linkage map

We genotyped a total of 417 flies, comprising 113 females and 104 males from the *CTN42* × *FORS4* cross, and 111 females and 89 males from the *FORS4* × *CTN42* cross. Of the initial 61 SEQUENOM assays designed, three were invariant in our mapping population and uninformative despite earlier checks (s10, s36, s37); these were removed and the remaining 58 were assigned to four linkage groups corresponding to the four chromosomes of *D. serrata* at a linkage LOD of 10.0 (X: 8 SNPs; Chr 2: 26 SNPs; Chr 3: 23 SNPs; and Chr 4: 1 SNP). We observed no differences in linkage group assignment between sexes or the two reciprocal crosses. No SNPs remained unassigned (Table 1; see File S1 for raw genotype data).

**Table 1:** EST derived SNP markers used to construct the *D. serrata* linkage map

Marker	*D. serrata* Linkage Group	*D. melanogaster* Chr. Arm	CG No.	Flybase Locus Name	FlyBaseID	MAF	*P* Value
s1	X	X	CG14792	Dmel\sta	FBgn0003517	0.45	0.000
s2	X	X	CG6186	Dmel\Tsf1	FBgn0022355	0.40	0.000
s3	3	3R	CG5887	Dmel\desat1	FBgn0086687	0.49	0.000
s4	2	2R	CG3401	Dmel\BTub60D	FBgn0003888	0.35	0.107
s5	3	3R	CG11522	Dmel\RpL6	FBgn0039857	0.26	0.012
s6	2	2L	CG7361	Dmel\RFeSP	FBgn0021906	0.43	0.001
s7	3	3R	CG2216	Dmel\Fer1HCH	FBgn0015222	0.43	0.000
s8	2	2L	CG13094	Dmel\Dh31	FBgn0032048	0.37	0.290
s9	2	2R	CG3124	Dmel\CG3124	FBgn0034840	0.29	0.000
s10	Not mapped	2L	CG9042	Dmel\Gpdh	FBgn0001128	0.00	0.000
s11	2*^a^*	2L	CG31811	Dmel\cenG1A	FBgn0028509	0.12	0.000
s12	2	2L	CG31811	Dmel\cenG1A	FBgn0028509	0.29	0.000
s13	3	3R	CG15697	Dmel\RpS30	FBgn0038834	0.31	0.546
s14	3	3L	CG6988	Dmel\Pdi	FBgn0014002	0.31	0.345
s15	3	3R	CG11901	Dmel\Ef1γ	FBgn0029176	0.33	0.000
s16	2	2L	CG34394	Dmel\CG34394	FBgn0085423	0.39	0.042
s17	3	3R	CG8036	Dmel\CG8036	FBgn0037607	0.29	0.046
s18	2	2L	CG9244	Dmel\Acon	FBgn0010100	0.46	0.000
s19	2	2R	CG6692	Dmel\Cp1	FBgn0013770	0.38	0.084
s20	3	3R	CG5502	Dmel\RpL4	FBgn0003279	0.29	0.138
s21	2	2L	CG6105	Dmel\I(2)06225	FBgn0010612	0.49	0.000
s22	3	3L	CG4769	Dmel\CG4769	FBgn0035600	0.31	0.478
s23	2*^a^*	2R	CG5330	Dmel\Nap1	FBgn0015268	0.18	0.000
s24	2	2R	CG5330	Dmel\Nap1	FBgn0015268	0.37	0.338
s25	3	3L	CG11276	RpS4	FBgn0011284	0.28	0.026
s26	3	3L	CG6871	Dmel\Cat	FBgn0000261	0.29	0.210
s27	3	3L	CG6871	Dmel\Cat	FBgn0000261	0.30	0.347
s28	X	X	CG8893	Dmel\Gapdh2	FBgn0001091	0.43	0.000
s29	X	X	CG14235	Dmel\CG14235	FBgn0031066	0.42	0.000
s30	3	3R	CG2216	Dmel\Fer1HCH	FBgn0015222	0.44	0.000
s31	3	3R	CG10901	Dmel\osk	FBgn0003015	0.30	0.482
s32	3	3R	CG11901	Dmel\Ef1γ	FBgn0029176	0.30	0.364
s33	X	X	CG1372	Dmel\yl	FBgn0004649	0.43	0.000
s34	2*^a^*	2R	CG3161	Dmel\Vha16-1	FBgn0262736	0.17	0.000
s35	3	3R	CG11033	Dmel\Kdm2	FBgn0037659	0.48	0.000
s36	Not mapped	3R	CG7610	Dmel\ATPsyn-γ	FBgn0020235	0.00	0.000
s37	Not mapped	3L	CG6988	Dmel\Pdi	FBgn0014002	0.00	0.000
s38	3	3L	CG4183	Dmel\Hsp26	FBgn0001225	0.29	0.061
s39	2	2L	CG5397	Dmel\CG5397	FBgn0031327	0.40	0.021
s40	X	X	CG32635	Dmel\CG32635	FBgn0052635	0.41	0.000
s41	2	2L	CG4824	Dmel\BicC	FBgn0000182	0.37	0.205
s42	2*^a^*	2R	CG9364	Dmel\Treh	FBgn0003748	0.22	0.000
s43	3	3L	CG10472	Dmel\CG10472	FBgn0035670	0.30	0.421
s44	4	4	No definition line found	1095281-1095303	**_**	0.32	0.226
s45	3	3L	CG6806	Dmel\Lsp2	FBgn0002565	0.31	0.507
s46	3	3L	CG11793	Dmel\Sod	FBgn0003462	0.29	0.065
s47	3	3L	CG8189	Dmel\ATPsyn-b	FBgn0019644	0.28	0.032
s48	3	3L	No definition line found	18976884-18976901	**_**	0.33	0.485
s49	2	2R	CG18067	Dmel\CG18067	FBgn0034512	0.37	0.113
s50	2	2L	CG4233	Dmel\Got2	FBgn0001125	0.46	0.000
s51	X	X	CG32816	Dmel\CG32816	FBgn0052816	0.46	0.000
s52	2	2R	CG4696	Dmel\Mp20	FBgn0002789	0.35	0.081
s53	2	2L	CG9042	Dmel\Gpdh	FBgn0001128	0.40	0.025
s54	2	2R	CG8983	Dmel\ERp60	FBgn0033663	0.40	0.017
s55	X	X	CG14792	Dmel\sta	FBgn0003517	0.45	0.000
s56	2	2L	CG9075	Dmel\eIF-4a	FBgn0001942	0.43	0.000
s57	2	2L	CG9075	Dmel\eIF-4a	FBgn0001942	0.36	0.213
s58	2	2L	CG31811	Dmel\cenG1A	FBgn0028509	0.47	0.000
s59	2	2R	CG10911	Dmel\CG10911	FBgn0034295	0.43	0.000
s60	3	3R	CG6439	Dmel\CG6439	FBgn0038922	0.32	0.807
s61	2	2L	CG3763	Dmel\Fbp2	FBgn0000640	0.37	0.279

Included are *D. melanogaster* gene names corresponding to the top BLAST hit of the full EST to the *D. melanogaster* genome.

a Markers that were assigned to chromosomal arms but not mapped on the linkage map because of transmission ratio distortion. MAF is the minor allele frequency in the entire dataset of 414 individuals and *P*-values correspond to χ^2^ for deviation from Mendelian expectations in the combined datasets (d.f. = 2 for autosomal markers and d.f. = 1 for X-linked markers).

As observed on the physical map, the linkage group assignments had tight correspondence with the chromosomal locations of the *D. melanogaster* genes, which further suggests arm-level gene conservation between the two species (Table 1). We observed one instance of a marker being assigned to a different chromosome as reflected by its best BLAST hit to *D. melanogaster*. The top *D. melanogaster* BLAST hit for the EST from which we designed this assay was *Gapdh1* (e-value = 6.45381e-54), which resides on 2R in *D. melanogaster*. However in *D. serrata* the s28 marker co-segregated with all of the X-linked markers. An absence of male heterozygotes in our F2 mapping population also strongly suggested that this marker is indeed X-linked in *D. serrata*. Closer inspection of the BLAST table for this marker indicated a near equal quality hit to *Gapdh2* (*e*-value *=* 2.55014e-53). This gene is X-linked in *D. melanogaster* and therefore may be the most likely ortholog of the marker. The strong conservation of genes within homologous chromosomal arms observed for *D. serrata* is entirely consistent with previous studies of the genus *Drosophila* (Sturtevant and Novitski 1941).

Despite some differences in the location, but not direction, of TRD in the reciprocal crosses, we observed very few differences between linkage maps constructed from each reciprocal and therefore we constructed the final linkage map on the combined data set. After removing highly distorted markers (MAF < 0.25 threshold) we were able to order 54 on our linkage map. From chromosome 2 we removed four assigned markers (s11, s23, s34, s42). No markers were removed from the third or X-chromosomes. The placement of markers along the two autosomes corresponded to the chromosomal arm assignments in *D. melanogaster*; we did not observe any instance of a marker moving chromosomal arms on the basis of its expected arm designation in *D. melanogaster* (Figure 3).

**Figure 4:**
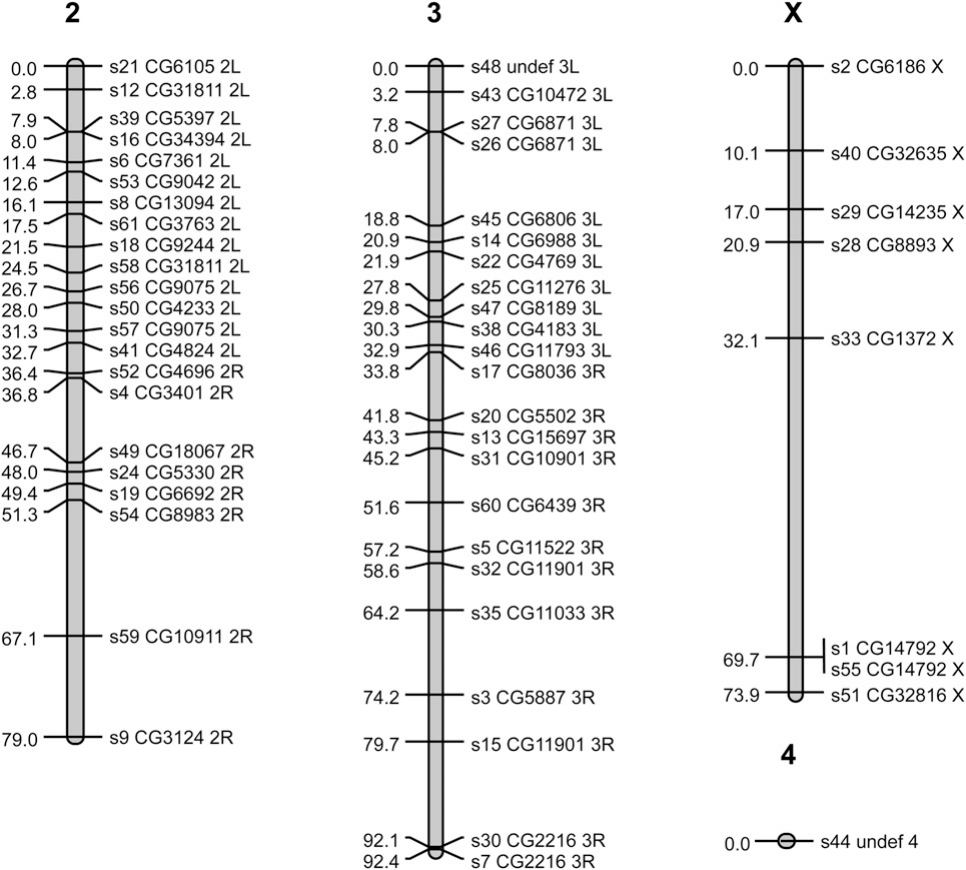
First-generation linkage map for *D. serrata*. Shown are the locations of EST-derived SNP markers on four chromosomes. SNP labels give the marker name followed by the *D. melanogaster* gene corresponding to the top BLAST hit of the EST from which the SNP assay was developed followed by the chromosomal arm on which it resides in *D. melanogaster*.

The total map length was 245.3 cM (Figure 4), which is similar to *D. melanogaster* (290 cM) (Catcheside 1977) but shorter than estimates reported for other species such as *D. pseudoobscura* (447cM) (Anderson 1990) or *D. simulans* (376.2 cm) (Barker and Moth 2001). At least two SNPs on the linkage map were physically mapped to each chromosome arm with the exception of the fourth (dot) chromosome, where only one SNP was assigned (Figures 1 and 2, blue text). Their positions on the physical map were comparable with their relative positions on the linkage map (Figure 3). The average marker coverage across the entire genome was one marker per 4.5 cM (X: 9.2 cM; Chr 2: 3.6 cM; Chr 3: 4.0 cM).

When we assayed two SNPs in the same gene, both usually mapped to the same location and were separated by very small distances (*e.g.*, s7 and s30, s26, and 27 and s1 and s55). However, in a few cases we observed discrepancies in the location of SNPs as determined by sequence homology with *D. melanogaster* and the linkage map. Markers s15 and s32 BLAST to different locations within the *Ef1γ* gene in *D. melanogaster*. Although both these SNPs mapped to 3L, they mapped to different locations along it, separated by approximately 21.1 cM. It is possible that there are multiple copies of the oligo binding sites for one of these assays in the *D. serrata* genome. A similar pattern was observed in two cases on 2L (s12 and s58, s57 and s56). Although it is also true that these cases of discrepancy in marker location may represent problems with the genotyping assay, because we cannot currently exclude the multiple copy hypothesis we have retained them on the map.

We observed significant TRD in the F2 crosses. When analyzing the reciprocals separately by sex, several of the autosomal markers showed significant deviations from Mendelian expectations at α = 0.05 (FORS4 × CTN42: males 48%, females 32%; CTN42 × FORS4: males 30%, females 28%). There was generally a large degree of overlap in the identity of distorted markers in the reciprocal crosses but there were also markers showing distortion in only one cross (Table S2). For X-linked markers, we only detected two instances of apparent but weak distortion in FORS4 × CTN42 (males for s28 and s29). We observed consistency in the direction of TRD with an overrepresentation of CTN 42 alleles in all but 2 of the 69 individual significant tests (Table S2).

This result suggests that the apparent distortion was not a consequence of genotyping error but instead reflects a biological phenomenon. TRD is a common observation in both interspecific and intraspecific *Drosophila* crosses and may indicate segregation distortion (Lyttle 1991), although its demonstration requires additional information. TRD is more common in interspecific than intraspecific crosses but may arise from genetic incompatibilities among populations. The Cooktown and Forster populations crossed in this study represent near-extreme ends of the natural distribution of *D. serrata* along the eastern coast of Australia and are separated by more than 2000 km. Previous work has shown that population structure tends to be weak in this species (Magiafoglou *et al.* 2002, Van Heerwaarden *et al.* 2009), but it does exhibit a pattern of isolation by-distance at neutral markers (Chenoweth and Blows 2008), suggesting that the evolution of gametic incompatibilities between distant populations of *D. serrata* is possible. Although chromosomal inversions are sometimes associated with TRD (Lyon 2003), we karyotyped the *CTN42* and *FORS4* lines before crossing and verified that they were homosequential and therefore are able to exclude large inversions as cause of the TRD. However, it remains possible that smaller inversions contributing to TRD were undetected by our screening procedures. The pattern could also be explained by the fixation of a greater number of deleterious mutations in the *FORS4* line during inbreeding as compared with *CTN42*. However, as many of these alleles would be expected to be recessive, this pattern predicts a deficiency of CTN42 homozygotes in the F2 population. The fact that in many cases we observed a deficiency of heterozygotes rather than homozygotes to some extent precludes this explanation, leaving genomic incompatibilities a more likely explanation for the observed pattern.

We have presented an extensive physical map and a first-generation linkage map for *D. serrata*, a model species for studying climatic adaptation and sexual selection, and we have provided a comparison of the location of genes with *D. melanogaster*. There was strong conservation of genes within chromosomal arms but a lack of macrosynteny within chromosomal arms. This work suggests that it will be relatively straightforward to assign genome scaffolds to chromosomal arms on the basis of homology with *D. melanogaster* but establishing scaffold order will remain challenging. However as in many species of *Drosophila*, where small regions of microsynteny are often highly conserved, phylogenetically-informed bioinformatic approaches (Bhutkar *et al.* 2006) can exploit this microsynteny to great effect to assign scaffold orders (Schaeffer *et al.* 2008). The linkage map presented here will be useful for initial QTL mapping, and as a basis for guiding marker development for fine mapping efforts.

## Supplementary Material

Supporting Information
