# Supplementary material for: Physical and Linkage Maps for Drosophila serrata, a Model Species for Studies of Clinal Adaptation and Sexual Selection
Source: G3 (Bethesda). 2012 Feb 1;2(2):287–97. doi: 10.1534/g3.111.001354 (PMC3284336; doi:10.1534/g3.111.001354)
Supplement: Supporting Information [file supp_2_2_287__index.html]

Supporting Information 

# Physical and Linkage Maps for *Drosophila serrata*, a Model Species for Studies of Clinal Adaptation and Sexual Selection

## Supporting Information for Stocker *et al.*, 2012

**Files in this Data Supplement:**

- Supporting Information - File S1 and Tables S1-S3 (PDF, 299 KB)
- Table S1 - Comparative Gene Locations in *D. melanogaster* and *D. serrata* (PDF, 183 KB)
- Table S2 - (PDF, 114 KB)
- Table S3 - Order of genetic markers along the chromosomes of *Drosophila* species (.xlsx, 23 KB)
- File S1 - F2 SNP Data Mapmaker Format (.zip, 8 KB)
