# Supplementary material for: Physical and Linkage Maps for Drosophila serrata, a Model Species for Studies of Clinal Adaptation and Sexual Selection
Source: G3 (Bethesda). 2012 Feb 1;2(2):287–97. doi: 10.1534/g3.111.001354 (PMC3284336; doi:10.1534/g3.111.001354)
Supplement: Supporting Information [file supp_2.2.287_001354SI.pdf]

Table S1 Comparative Gene Locations in *D. melanogaster* and *D. serrata*

Chromosome X (Muller element A)

Gene	Dm Flybase code	*D. mel* location	*D. serrata* location
SNP s55<br>stubarista (sta)	CG14792	X2B1	X2B
Ds CL472Contig1<br>protein coding gene	CG8128	X13E7-8	X5A
ATP7	CG1886	X10F1-2	X5B
lethal(1) G0196<br>(l(1) G0196)	CG14616	X20C1	X5D (~same as stn A)
stoned A (stn A)	CG12500	X20F3	X5D (prox to 14616)
Actin (Act5C)	CG4027	X5C7	X6C
SNP s33<br>yolkless (yl)	CG1372	X12E3	X7A
Ds CL1017Contig1<br>CHOp24	CG3564	X4C7	X8D
Ds CL49Contig1<br>Autophagy specif<br>Gene 8a (Atg8a)	CG32672	X9E6-7	X11E
SNP s29<br>protein coding gene	CG14235	X18E5	X12C
Ds CL0Est<br>annexin (AnnX)	CG9579	X19C1	X14B
white (w)	CG2759	X3B6	X15A(from pUAST)

Chromosome Dm 2L (Muller element B)

Gene	Dm Flybase code	Dm Location	Ds location
Ds Clone4994573C12<br>protein coding gene	CG17633	2L~30C8	2L24A
synthesis of cyto-<br>chrome c oxidase (sco1)	CG8885	2L25B5	2L25A
synaptotagmin1<br>(Syt 1)	CG3139	2L23A6-B1	2L25C
SNP s53<br>Glycerol-3-phosphate<br>dehydrogenase (Gpdh)	CG9042	2L26A3	2L26A
Ds Clone4952873C05<br>Imaginal disc growth<br>factor 1 (Idgf1)	CG4472	2L36A1	2L33C

donut (dnt)	CG17559	2L37D2	2L35B
Ds Clone4982073E19 Larval serum protein 1 β (Lsp1β)	CG4178	2L21E2	2L35C
SNP s56 Eukaryote initiation factor 4a (eIF-4a)	CG9075	2L26B2	2L36B
black (b)	CG7811	2L34D1	2L39C

Chromosome 2R (Muller element C)

Gene	Dm Flybase code	Dm Location	Ds location
Actin (Act42A)	CG12051	2R42A7	2R41A
Ds CL589Contig1 Heat shock protein cognate 5 (Hsc70-5)	CG8542	2R50E6	2R44B
protein coding gene	CG8740	2R44E3-4	2R44B
SNP s4 β tubulin 60D* (βTub60D)	CG3401	2R60C6	2R45A
SNP s52 Muscle protein 20 (Mp20)	CG4696	2R49F13	2R45A
Ds CL1086Contig1 protein coding gene	CG8736	2R44D4	2R45C
Ribosomal protein LP2 (RpLP2)	CG4918	2R53C9	2R46A
β tubulin (βTub56D)	CG9277	2R56D7-8	2R47B
trplike (trpl)	CG18345	2R46B2	2R48B
even skipped (eve)	CG2328	2R46C10	2R49A
Actin (Act57B)	CG10067	2R57B5	2R50B
morula (mr)	CG3060	2R60A4	2R53A
Ds CL388Contig1 protein coding gene	CG4019	2R59F4	2R56C
engrailed (en)	CG9015	2R47F17	2R57C
Ds CL1069Contig1 Trehalose transporter 1-1 Tret1-1	CG30035	2R48B6-7	2R57D
Ds CL1004Contig1 potein coding gene	CG12736	2R43D1	2R59A
kruppel (kr)	CG3340	2R60F6	2R60C (near telomere)

Chromosome 3L (Muller element D)

Gene	Dm Flybase code	Dm Location	Ds location
protein coding gene	CG7376	3L65A5	3L61C
ecdy. induced prot. (Eip74EF)	CG32180	3L74D4-E2	3L62B
SNP s26, Catalase (Cat)	CG6871	3L75E1	3L62C
Ds CL230(203?)Contig1 protein coding gene	CG3819	3L75E6	3L63B
Ds CL159Contig1, UDP-galactose 4'-epimerase (Gale)	CG12030	3L61C8	3L65A
Ds CL265Contig1 protein coding gene	CG14997	3L64A10	3L65C
ecdy. induced prot. (Eip75B)	CG8127	3L75A10-B6	3L67C
heat shock prot. 83 (Hsp83)	CG1242	3L63B11	3L68D
knirps (kni)	CG4717	3L77E3	3L71B
Actin (Act79B)	CG7478	3L79B	3L72
SNP s47, ATP synthase, subunit b ATPsyn-b	CG8189	3L67C5	3L74C
eukar init. Factor 4E (eIF-4E)	CG4035	3L67B4	3L75B
Ds CL231Contig1 Astray (aay)	CG3705	3L67B5	3L75C
α tubulin (αTub67C)	CG8308	3L67C4	3L75C
Ds CL920Contig1 tumor sup prot 101 (TSG101)	CG9712	3L73D1	3L76A

Chromosome 3R (Muller element E)

Gene	Dm Flybase code	Dm Location	Ds location
Ds CL0EstAϕ4 Regena (Rga)	CG2161	3R83B5-6	3R81A
Senescense marker prot. 30 (smp-30)	CG7390	3R88D2	3R82D
Actin (Act88F)	CG5178	3R88F	3R83
hunchback (hb)	CG9786	3R85A5	3R83D

α tubulin (αTub85E)	CG9476	3R85E6	3R84A
Heat shock RNAω (Hsrω)	CR31400	3R93D4	3R84A
SNP s20, Ribosomal Protein L4 (RpL4)	CG5502	3R98B6	3R85B
Ds CL0EstOϕ3 Elongin B (Elongin-B)	CG4204	3R92F2	3R86A
Glutamate de-hydrogenase (Gdh)	CG5320	3R95C13-D1	3R86C
Heat shock prot. 68 (Hsp68)	CG5436	3R95D11	3R86
Antennapedia (Antp)	CG1028	3R84A6	3R87C
α tubulin (αTub84B)	CG1913	3R84B2	3R87D
α tubulin (αTub84D)	CG2512	3R84D9	3R87D
Actin (Act88F)	CG5178	3R88F	3R88
Ds CL533Contig1 myosin alk lite ch (Mlc1)	CG5596	3R98A14-15	3R89C
β tubulin (βTub85D)	CG9359	3R85D15	3R92
transient receptor potential (trp)	CG7875	3R99C6-G7	3R93A
β tubulin (βTub97EF)	CG4869	3R98B6-7	3R93
Heat shock prot. 70 (Hsp70)	5, depends on probe used	3R87A2-3/ B12-15	3R94
Actin (Act87E)	CG18290	3R87E	3R95D
globin1 (glob1)	CG9734	3R89A8	3R96A
Ds CL22Contig1 Aldolase (Ald)	CG6058	3R97A6	3R96B
SNP s7, Ferritin 1 heavy chain homolog (Fer1HCH)	CG2216	3R99F2	3R97B
Ribosomal protein L32 (rp49, RpL32)	CG7939	3R99D3	3R100D (near telomere)

Chromosome 4 (Dm 4) (Muller element F)

**Gene**	**Dm Flybase code**	**Dm Location**	**Ds location**
Plasma membrane Calcium ATPase (PMCA)	CG42314	(4)102B	(4)102A

Table S2

Reciprocal cross									CTN42 x FORS4												FORS4 x CTN42					
Sex						Males						Females						Males						Females		
SNP Marker	*D. serrata* linkage group	d.f.	a	h	b	missing	χ2	p	a	h	b	missing	χ2	p	a	h	b	missing	χ2	p	a	h	b	missing	χ2	p
s1	X	1	58	0	46	0	1.38	0.239	52	61	0	1	0.72	0.397	50	0	37	2	1.94	0.163	0	53	55	3	0.04	0.847
s2	X	1	45	0	59	0	1.88	0.170	54	59	0	1	0.22	0.638	42	0	45	2	0.10	0.748	0	54	53	4	0.01	0.923
s3	3	2	47	42	15	0	23.54	0.000	40	55	18	1	8.65	0.013	32	45	10	2	11.23	0.004	33	60	17	1	5.56	0.062
s4	2	2	29	54	20	1	1.82	0.403	27	61	25	1	0.79	0.674	26	48	13	2	4.82	0.090	24	59	27	1	0.75	0.689
s5	3	2	24	51	23	6	0.18	0.912	20	51	35	7	4.40	0.111	18	42	23	6	0.61	0.735	12	50	34	15	10.25	0.006
s6	2	2	28	50	25	1	0.26	0.877	33	52	28	1	1.16	0.560	34	39	14	2	10.13	0.006	42	44	24	1	10.29	0.006
s7	3	2	49	43	11	1	30.84	0.000	33	62	18	1	5.05	0.080	45	37	5	2	38.72	0.000	54	48	8	1	40.25	0.000
s8	2	2	25	53	26	0	0.06	0.972	29	56	28	1	0.03	0.987	30	42	15	2	5.28	0.072	33	50	27	1	1.56	0.458
s9	2	2	72	26	6	0	109.77	0.000	75	34	4	1	107.14	0.000	47	32	8	2	41.05	0.000	51	47	12	1	29.98	0.000
s10	Invariant		104	0	0	0			113	0	0	1			87	0	0	2			110	0	0	1		
s11	2	2	78	25	1	0	142.06	0.000	93	20	0	1	200.24	0.000	72	15	0	2	156.52	0.000	85	25	0	1	164.09	0.000
s12	2	2	62	17	25	0	73.44	0.000	82	6	25	1	147.78	0.000	64	8	15	2	113.14	0.000	71	14	25	1	99.60	0.000
s13	3	2	28	51	19	6	1.82	0.403	20	62	22	9	3.92	0.141	15	38	26	10	3.18	0.204	22	45	26	18	0.44	0.802
s14	3	2	30	53	21	0	1.60	0.450	26	53	34	1	1.57	0.457	17	49	21	2	1.76	0.415	20	66	24	1	4.69	0.096
s15	3	2	66	17	18	3	90.07	0.000	62	17	32	3	69.63	0.000	54	15	13	7	73.98	0.000	68	11	29	3	96.65	0.000
s16	2	2	27	54	23	0	0.46	0.794	32	54	27	1	0.66	0.718	32	40	14	3	7.95	0.019	30	56	21	4	1.75	0.417
s17	3	2	28	56	20	0	1.85	0.397	21	61	31	1	2.49	0.288	15	49	23	2	2.86	0.239	22	65	23	1	3.65	0.161
s18	2	2	39	53	12	0	14.06	0.001	45	61	7	1	26.27	0.000	39	38	10	2	20.72	0.000	48	45	17	1	21.11	0.000
s19	2	2	33	48	23	0	2.54	0.281	24	63	26	1	1.57	0.457	32	42	13	2	8.40	0.015	30	55	25	1	0.45	0.797
s20	3	2	23	58	21	2	2.00	0.368	22	68	22	2	5.14	0.076	17	42	26	4	1.92	0.383	25	54	29	3	0.30	0.862
s21	2	2	36	50	18	0	6.38	0.041	40	54	19	1	8.03	0.018	39	40	8	2	22.66	0.000	48	50	12	1	24.47	0.000
s22	3	2	31	51	22	0	1.60	0.450	26	55	32	1	0.72	0.699	16	49	22	2	2.22	0.330	21	63	26	1	2.78	0.249
s23	2	2	80	21	1	2	157.67	0.000	81	30	1	2	138.43	0.000	59	27	1	2	89.85	0.000	72	31	6	2	100.19	0.000
s24	2	2	30	50	24	0	0.85	0.655	23	61	29	1	1.35	0.508	31	44	12	2	8.31	0.016	29	54	27	1	0.11	0.947
s25	3	2	29	54	21	0	1.38	0.500	19	64	30	1	4.13	0.127	14	52	21	2	4.45	0.108	21	62	27	1	2.44	0.296
s26	3	2	18	61	25	0	4.06	0.131	33	53	27	1	1.07	0.585	15	51	21	2	3.41	0.181	23	57	30	1	1.04	0.596
s27	3	2	19	60	25	0	3.15	0.207	33	53	27	1	1.07	0.585	15	51	21	2	3.41	0.181	25	56	29	1	0.33	0.849
s28	X	1	49	0	54	1	0.24	0.622	50	63	0	1	1.50	0.221	55	0	32	2	6.08	0.014	0	50	60	1	0.91	0.340
s29	X	1	45	0	59	0	1.88	0.170	52	61	0	1	0.72	0.397	53	0	34	2	4.15	0.042	0	49	59	3	0.93	0.336
s30	3	2	49	44	11	0	30.23	0.000	33	62	18	1	5.05	0.080	45	37	5	2	38.72	0.000	52	49	8	2	36.63	0.000
s31	3	2	28	53	23	0	0.52	0.771	21	68	23	2	5.21	0.074	20	41	26	2	1.11	0.573	24	54	32	1	1.20	0.549
s32	3	2	30	52	22	0	1.23	0.540	22	57	34	1	2.56	0.278	21	43	23	2	0.10	0.950	19	58	33	1	3.89	0.143
s33	X	1	50	0	54	0	0.15	0.695	55	58	0	1	0.08	0.778	49	0	38	2	1.39	0.238	0	50	60	1	0.91	0.340
s34	2	2	81	18	5	0	155.54	0.000	84	24	5	1	147.85	0.000	62	25	0	2	104.10	0.000	75	34	1	1	115.60	0.000
s35	3	2	47	49	7	1	31.31	0.000	53	51	8	2	37.05	0.000	25	48	14	2	3.71	0.156	36	55	19	1	5.25	0.072
s36	Invariant		104	0	0	0			113	0	0	1			87	0	0	2			110	0	0	1		
s37	Invariant		104	0	0	0			112	0	0	2			87	0	0	2			109	0	0	2		
s38	3	2	30	53	20	1	2.03	0.363	19	64	30	1	4.13	0.127	16	48	23	2	2.06	0.357	21	64	25	1	3.24	0.198
s39	2	2	28	53	23	0	0.52	0.771	32	55	26	1	0.72	0.699	33	40	14	2	8.86	0.012	31	58	21	1	2.15	0.342
s40	X	1	46	0	58	0	1.38	0.239	49	64	0	1	1.99	0.158	50	0	37	2	1.94	0.163	0	50	59	2	0.74	0.389
s41	2	2	26	55	23	0	0.52	0.771	31	56	26	1	0.45	0.798	28	44	15	2	3.90	0.143	29	56	25	1	0.33	0.849
s42	2	2	80	18	6	0	149.77	0.000	84	24	5	1	147.85	0.000	53	32	2	2	65.87	0.000	59	50	1	1	62.07	0.000
s43	3	2	24	54	26	0	0.23	0.891	32	53	28	1	0.72	0.699	15	53	19	2	4.52	0.104	21	56	33	1	2.65	0.265
s44	4	2	29	54	21	0	1.38	0.500	27	58	27	2	0.14	0.931	22	49	16	2	2.22	0.330	17	63	30	1	5.40	0.067
s45	3	2	31	51	22	0	1.60	0.450	24	56	33	1	1.44	0.486	19	48	20	2	0.95	0.621	20	62	28	1	2.95	0.229
s46	3	2	28	56	20	0	1.85	0.397	21	60	31	2	2.36	0.308	16	47	24	2	2.03	0.362	20	64	24	3	4.00	0.135
s47	3	2	29	52	21	2	1.29	0.524	19	65	29	1	4.33	0.115	15	50	22	2	3.07	0.216	21	64	25	1	3.24	0.198
s48	3	2	23	54	27	0	0.46	0.794	31	54	28	1	0.38	0.827	17	53	16	3	4.67	0.097	27	56	24	4	0.40	0.818
s49	2	2	30	53	21	0	1.60	0.450	24	62	27	1	1.23	0.541	30	44	12	3	7.58	0.023	31	53	26	1	0.60	0.741
s50	2	2	32	51	21	0	2.37	0.306	33	59	21	1	2.77	0.250	36	43	8	2	18.03	0.000	44	49	17	1	14.56	0.001
s51	X	1	61	0	42	1	3.50	0.061	54	58	0	2	0.14	0.705	50	0	37	2	1.94	0.163	0	50	59	2	0.74	0.389
s52	2	2	29	55	20	0	1.90	0.386	27	61	25	1	0.79	0.674	25	49	13	2	4.70	0.095	26	58	26	1	0.33	0.849
s53	2	2	27	50	26	1	0.11	0.948	33	52	28	1	1.16	0.560	34	36	17	2	9.23	0.010	33	50	27	1	1.56	0.458
s54	2	2	36	44	24	0	5.23	0.073	30	61	22	1	1.85	0.397	33	39	15	2	8.38	0.015	27	56	25	3	0.22	0.895
s55	X	1	58	0	46	0	1.38	0.239	52	61	0	1	0.72	0.397	50	0	37	2	1.94	0.163	0	54	54	3	0.00	1.000
s56	2	2	30	53	21	0	1.60	0.450	33	58	22	1	2.22	0.329	33	43	13	0	9.09	0.011	39	53	18	1	8.16	0.017
s57	2	2	24	56	24	0	0.62	0.735	30	59	24	1	0.86	0.651	26	46	17	0	1.92	0.383	29	56	25	1	0.33	0.849
s58	2	2	44	42	15	3	19.51	0.000	35	62	11	5	13.04	0.001	37	41	9	2	18.31	0.000	42	49	11	9	19.00	0.000
s59	2	2	58	37	9	0	54.83	0.000	63	42	8	1	60.98	0.000	32	46	11	0	10.01	0.007	32	60	18	1	4.47	0.107
s60	3	2	28	53	23	0	0.52	0.771	21	70	23	0	6.00	0.050	23	42	24	0	0.30	0.859	27	46	37	1	4.76	0.092
s61	2	2	27	53	24	0	0.21	0.900	29	59	26	0	0.30	0.861	30	41	18	0	3.79	0.151	29	55	26	1	0.16	0.921

Table S3 Order of genetic markers along the chromosomes of *Drosophila* species. The locations of sequence orthologous to those used for the D. serrata physical map were found for the Drosophila species previously sequenced in the 12 Genomes Project (Shaeffer et al. 2008) by a manual search on Flybase. The genes are ordered according to their relative positions along the chromosome arms. Spacing between genes in this table is not proportional to their spacing in the genome. The *D. melanogaster* region numbers for each chromosome were used to orient the marker sequences in melanogaster group species and also in D. serrata, which has the same region numbering order of its chromosome arms as *D. melanogaster*. The CG numbers for *D. melanogaster* were used in the comparisons. The centromeres are indicated by black boxes and fixed inversions between related species are outlined in black. Translocations/ transpositions are also outlined in black.

Table S3 is available for download at http://www.g3journal.org/lookup/suppl/doi:10.1534/g3.111.001354/-/DC1 as an Excel file.

File S1 F2 SNP Data Mapmaker Format

File S1 is available for download at http://www.g3journal.org/lookup/suppl/doi:10.1534/g3.111.001354/-/DC1 as a zip file.
