# Supplementary material for: Physical and Linkage Maps for Drosophila serrata, a Model Species for Studies of Clinal Adaptation and Sexual Selection
Source: G3 (Bethesda). 2012 Feb 1;2(2):287–97. doi: 10.1534/g3.111.001354 (PMC3284336; doi:10.1534/g3.111.001354)
Supplement: Supporting Information [file supp_2.2.287_TableS1.pdf]

Table S1 Comparative Gene Locations in *D. melanogaster* and *D. serrata*

Chromosome X (Muller element A)

Gene	Dm Flybase code	*D. mel* location	*D. serrata* location
SNP s55<br>stubarista (sta)	CG14792	X2B1	X2B
Ds CL472Contig1<br>protein coding gene	CG8128	X13E7-8	X5A
ATP7	CG1886	X10F1-2	X5B
lethal(1) G0196<br>(l(1) G0196)	CG14616	X20C1	X5D (~same as stn A)
stoned A (stn A)	CG12500	X20F3	X5D (prox to 14616)
Actin (Act5C)	CG4027	X5C7	X6C
SNP s33<br>yolkless (yl)	CG1372	X12E3	X7A
Ds CL1017Contig1<br>CHOp24	CG3564	X4C7	X8D
Ds CL49Contig1<br>Autophagy specif<br>Gene 8a (Atg8a)	CG32672	X9E6-7	X11E
SNP s29<br>protein coding gene	CG14235	X18E5	X12C
Ds CL0Est<br>annexin (AnnX)	CG9579	X19C1	X14B
white (w)	CG2759	X3B6	X15A(from pUAST)

Chromosome Dm 2L (Muller element B)

Gene	Dm Flybase code	Dm Location	Ds location
Ds Clone4994573C12<br>protein coding gene	CG17633	2L~30C8	2L24A
synthesis of cyto-<br>chrome c oxidase (sco1)	CG8885	2L25B5	2L25A
synaptotagmin1<br>(Syt 1)	CG3139	2L23A6-B1	2L25C
SNP s53<br>Glycerol-3-phosphate<br>dehydrogenase (Gpdh)	CG9042	2L26A3	2L26A
Ds Clone4952873C05<br>Imaginal disc growth<br>factor 1 (Idgf1)	CG4472	2L36A1	2L33C

donut (dnt)	CG17559	2L37D2	2L35B
Ds Clone4982073E19 Larval serum protein 1 β (Lsp1β)	CG4178	2L21E2	2L35C
SNP s56 Eukaryote initiation factor 4a (eIF-4a)	CG9075	2L26B2	2L36B
black (b)	CG7811	2L34D1	2L39C

Chromosome 2R (Muller element C)

Gene	Dm Flybase code	Dm Location	Ds location
Actin (Act42A)	CG12051	2R42A7	2R41A
Ds CL589Contig1 Heat shock protein cognate 5 (Hsc70-5)	CG8542	2R50E6	2R44B
protein coding gene	CG8740	2R44E3-4	2R44B
SNP s4 β tubulin 60D* (βTub60D)	CG3401	2R60C6	2R45A
SNP s52 Muscle protein 20 (Mp20)	CG4696	2R49F13	2R45A
Ds CL1086Contig1 protein coding gene	CG8736	2R44D4	2R45C
Ribosomal protein LP2 (RpLP2)	CG4918	2R53C9	2R46A
β tubulin (βTub56D)	CG9277	2R56D7-8	2R47B
trplike (trpl)	CG18345	2R46B2	2R48B
even skipped (eve)	CG2328	2R46C10	2R49A
Actin (Act57B)	CG10067	2R57B5	2R50B
morula (mr)	CG3060	2R60A4	2R53A
Ds CL388Contig1 protein coding gene	CG4019	2R59F4	2R56C
engrailed (en)	CG9015	2R47F17	2R57C
Ds CL1069Contig1 Trehalose transporter 1-1 Tret1-1	CG30035	2R48B6-7	2R57D
Ds CL1004Contig1 potein coding gene	CG12736	2R43D1	2R59A
kruppel (kr)	CG3340	2R60F6	2R60C (near telomere)

Chromosome 3L (Muller element D)

Gene	Dm Flybase code	Dm Location	Ds location
protein coding gene	CG7376	3L65A5	3L61C
ecdy. induced prot. (Eip74EF)	CG32180	3L74D4-E2	3L62B
SNP s26, Catalase (Cat)	CG6871	3L75E1	3L62C
Ds CL230(203?)Contig1 protein coding gene	CG3819	3L75E6	3L63B
Ds CL159Contig1, UDP-galactose 4'-epimerase (Gale)	CG12030	3L61C8	3L65A
Ds CL265Contig1 protein coding gene	CG14997	3L64A10	3L65C
ecdy. induced prot. (Eip75B)	CG8127	3L75A10-B6	3L67C
heat shock prot. 83 (Hsp83)	CG1242	3L63B11	3L68D
knirps (kni)	CG4717	3L77E3	3L71B
Actin (Act79B)	CG7478	3L79B	3L72
SNP s47, ATP synthase, subunit b ATPsyn-b	CG8189	3L67C5	3L74C
eukar init. Factor 4E (eIF-4E)	CG4035	3L67B4	3L75B
Ds CL231Contig1 Astray (aay)	CG3705	3L67B5	3L75C
α tubulin (αTub67C)	CG8308	3L67C4	3L75C
Ds CL920Contig1 tumor sup prot 101 (TSG101)	CG9712	3L73D1	3L76A

Chromosome 3R (Muller element E)

Gene	Dm Flybase code	Dm Location	Ds location
Ds CL0EstAϕ4 Regena (Rga)	CG2161	3R83B5-6	3R81A
Senescense marker prot. 30 (smp-30)	CG7390	3R88D2	3R82D
Actin (Act88F)	CG5178	3R88F	3R83
hunchback (hb)	CG9786	3R85A5	3R83D

α tubulin (αTub85E)	CG9476	3R85E6	3R84A
Heat shock RNAω (Hsrω)	CR31400	3R93D4	3R84A
SNP s20, Ribosomal Protein L4 (RpL4)	CG5502	3R98B6	3R85B
Ds CL0EstOϕ3 Elongin B (Elongin-B)	CG4204	3R92F2	3R86A
Glutamate de-hydrogenase (Gdh)	CG5320	3R95C13-D1	3R86C
Heat shock prot. 68 (Hsp68)	CG5436	3R95D11	3R86
Antennapedia (Antp)	CG1028	3R84A6	3R87C
α tubulin (αTub84B)	CG1913	3R84B2	3R87D
α tubulin (αTub84D)	CG2512	3R84D9	3R87D
Actin (Act88F)	CG5178	3R88F	3R88
Ds CL533Contig1 myosin alk lite ch (Mlc1)	CG5596	3R98A14-15	3R89C
β tubulin (βTub85D)	CG9359	3R85D15	3R92
transient receptor potential (trp)	CG7875	3R99C6-G7	3R93A
β tubulin (βTub97EF)	CG4869	3R98B6-7	3R93
Heat shock prot. 70 (Hsp70)	5, depends on probe used	3R87A2-3/ B12-15	3R94
Actin (Act87E)	CG18290	3R87E	3R95D
globin1 (glob1)	CG9734	3R89A8	3R96A
Ds CL22Contig1 Aldolase (Ald)	CG6058	3R97A6	3R96B
SNP s7, Ferritin 1 heavy chain homolog (Fer1HCH)	CG2216	3R99F2	3R97B
Ribosomal protein L32 (rp49, RpL32)	CG7939	3R99D3	3R100D (near telomere)

Chromosome 4 (Dm 4) (Muller element F)

Gene	Dm Flybase code	Dm Location	Ds location
Plasma membrane Calcium ATPase (PMCA)	CG42314	(4)102B	(4)102A
