# Supplementary material for: Physical and Linkage Maps for Drosophila serrata, a Model Species for Studies of Clinal Adaptation and Sexual Selection
Source: G3 (Bethesda). 2012 Feb 1;2(2):287–97. doi: 10.1534/g3.111.001354 (PMC3284336; doi:10.1534/g3.111.001354)
Supplement: Supporting Information [file supp_2.2.287_TableS2.pdf]

Table S2

Reciprocal cross			CTN42 x FORS4												FORS4 x CTN42											
Sex			Males						Females						Males						Females					
SNP Marker	*D. serrata* linkage group	d.f.	a	h	b	missing	χ2	p	a	h	b	missing	χ2	p	a	h	b	missing	χ2	p	a	h	b	missing	χ2	p
s1	X	1	58	0	46	0	1.38	0.239	52	61	0	1	0.72	0.397	50	0	37	2	1.94	0.163	0	53	55	3	0.04	0.847
s2	X	1	45	0	59	0	1.88	0.170	54	59	0	1	0.22	0.638	42	0	45	2	0.10	0.748	0	54	53	4	0.01	0.923
s3	3	2	47	42	15	0	23.54	0.000	40	55	18	1	8.65	0.013	32	45	10	2	11.23	0.004	33	60	17	1	5.56	0.062
s4	2	2	29	54	20	1	1.82	0.403	27	61	25	1	0.79	0.674	26	48	13	2	4.82	0.090	24	59	27	1	0.75	0.689
s5	3	2	24	51	23	6	0.18	0.912	20	51	35	7	4.40	0.111	18	42	23	6	0.61	0.735	12	50	34	15	10.25	0.006
s6	2	2	28	50	25	1	0.26	0.877	33	52	28	1	1.16	0.560	34	39	14	2	10.13	0.006	42	44	24	1	10.29	0.006
s7	3	2	49	43	11	1	30.84	0.000	33	62	18	1	5.05	0.080	45	37	5	2	38.72	0.000	54	48	8	1	40.25	0.000
s8	2	2	25	53	26	0	0.06	0.972	29	56	28	1	0.03	0.987	30	42	15	2	5.28	0.072	33	50	27	1	1.56	0.458
s9	2	2	72	26	6	0	109.77	0.000	75	34	4	1	107.14	0.000	47	32	8	2	41.05	0.000	51	47	12	1	29.98	0.000
s10	Invariant		104	0	0	0			113	0	0	1			87	0	0	2			110	0	0	1		
s11	2	2	78	25	1	0	142.06	0.000	93	20	0	1	200.24	0.000	72	15	0	2	156.52	0.000	85	25	0	1	164.09	0.000
s12	2	2	62	17	25	0	73.44	0.000	82	6	25	1	147.78	0.000	64	8	15	2	113.14	0.000	71	14	25	1	99.60	0.000
s13	3	2	28	51	19	6	1.82	0.403	20	62	22	9	3.92	0.141	15	38	26	10	3.18	0.204	22	45	26	18	0.44	0.802
s14	3	2	30	53	21	0	1.60	0.450	26	53	34	1	1.57	0.457	17	49	21	2	1.76	0.415	20	66	24	1	4.69	0.096
s15	3	2	66	17	18	3	90.07	0.000	62	17	32	3	69.63	0.000	54	15	13	7	73.98	0.000	68	11	29	3	96.65	0.000
s16	2	2	27	54	23	0	0.46	0.794	32	54	27	1	0.66	0.718	32	40	14	3	7.95	0.019	30	56	21	4	1.75	0.417
s17	3	2	28	56	20	0	1.85	0.397	21	61	31	1	2.49	0.288	15	49	23	2	2.86	0.239	22	65	23	1	3.65	0.161
s18	2	2	39	53	12	0	14.06	0.001	45	61	7	1	26.27	0.000	39	38	10	2	20.72	0.000	48	45	17	1	21.11	0.000
s19	2	2	33	48	23	0	2.54	0.281	24	63	26	1	1.57	0.457	32	42	13	2	8.40	0.015	30	55	25	1	0.45	0.797
s20	3	2	23	58	21	2	2.00	0.368	22	68	22	2	5.14	0.076	17	42	26	4	1.92	0.383	25	54	29	3	0.30	0.862
s21	2	2	36	50	18	0	6.38	0.041	40	54	19	1	8.03	0.018	39	40	8	2	22.66	0.000	48	50	12	1	24.47	0.000
s22	3	2	31	51	22	0	1.60	0.450	26	55	32	1	0.72	0.699	16	49	22	2	2.22	0.330	21	63	26	1	2.78	0.249
s23	2	2	80	21	1	2	157.67	0.000	81	30	1	2	138.43	0.000	59	27	1	2	89.85	0.000	72	31	6	2	100.19	0.000
s24	2	2	30	50	24	0	0.85	0.655	23	61	29	1	1.35	0.508	31	44	12	2	8.31	0.016	29	54	27	1	0.11	0.947
s25	3	2	29	54	21	0	1.38	0.500	19	64	30	1	4.13	0.127	14	52	21	2	4.45	0.108	21	62	27	1	2.44	0.296
s26	3	2	18	61	25	0	4.06	0.131	33	53	27	1	1.07	0.585	15	51	21	2	3.41	0.181	23	57	30	1	1.04	0.596
s27	3	2	19	60	25	0	3.15	0.207	33	53	27	1	1.07	0.585	15	51	21	2	3.41	0.181	25	56	29	1	0.33	0.849
s28	X	1	49	0	54	1	0.24	0.622	50	63	0	1	1.50	0.221	55	0	32	2	6.08	0.014	0	50	60	1	0.91	0.340
s29	X	1	45	0	59	0	1.88	0.170	52	61	0	1	0.72	0.397	53	0	34	2	4.15	0.042	0	49	59	3	0.93	0.336
s30	3	2	49	44	11	0	30.23	0.000	33	62	18	1	5.05	0.080	45	37	5	2	38.72	0.000	52	49	8	2	36.63	0.000
s31	3	2	28	53	23	0	0.52	0.771	21	68	23	2	5.21	0.074	20	41	26	2	1.11	0.573	24	54	32	1	1.20	0.549
s32	3	2	30	52	22	0	1.23	0.540	22	57	34	1	2.56	0.278	21	43	23	2	0.10	0.950	19	58	33	1	3.89	0.143
s33	X	1	50	0	54	0	0.15	0.695	55	58	0	1	0.08	0.778	49	0	38	2	1.39	0.238	0	50	60	1	0.91	0.340
s34	2	2	81	18	5	0	155.54	0.000	84	24	5	1	147.85	0.000	62	25	0	2	104.10	0.000	75	34	1	1	115.60	0.000
s35	3	2	47	49	7	1	31.31	0.000	53	51	8	2	37.05	0.000	25	48	14	2	3.71	0.156	36	55	19	1	5.25	0.072
s36	Invariant		104	0	0	0			113	0	0	1			87	0	0	2			110	0	0	1		
s37	Invariant		104	0	0	0			112	0	0	2			87	0	0	2			109	0	0	2		
s38	3	2	30	53	20	1	2.03	0.363	19	64	30	1	4.13	0.127	16	48	23	2	2.06	0.357	21	64	25	1	3.24	0.198
s39	2	2	28	53	23	0	0.52	0.771	32	55	26	1	0.72	0.699	33	40	14	2	8.86	0.012	31	58	21	1	2.15	0.342
s40	X	1	46	0	58	0	1.38	0.239	49	64	0	1	1.99	0.158	50	0	37	2	1.94	0.163	0	50	59	2	0.74	0.389
s41	2	2	26	55	23	0	0.52	0.771	31	56	26	1	0.45	0.798	28	44	15	2	3.90	0.143	29	56	25	1	0.33	0.849
s42	2	2	80	18	6	0	149.77	0.000	84	24	5	1	147.85	0.000	53	32	2	2	65.87	0.000	59	50	1	1	62.07	0.000
s43	3	2	24	54	26	0	0.23	0.891	32	53	28	1	0.72	0.699	15	53	19	2	4.52	0.104	21	56	33	1	2.65	0.265
s44	4	2	29	54	21	0	1.38	0.500	27	58	27	2	0.14	0.931	22	49	16	2	2.22	0.330	17	63	30	1	5.40	0.067
s45	3	2	31	51	22	0	1.60	0.450	24	56	33	1	1.44	0.486	19	48	20	2	0.95	0.621	20	62	28	1	2.95	0.229
s46	3	2	28	56	20	0	1.85	0.397	21	60	31	2	2.36	0.308	16	47	24	2	2.03	0.362	20	64	24	3	4.00	0.135
s47	3	2	29	52	21	2	1.29	0.524	19	65	29	1	4.33	0.115	15	50	22	2	3.07	0.216	21	64	25	1	3.24	0.198
s48	3	2	23	54	27	0	0.46	0.794	31	54	28	1	0.38	0.827	17	53	16	3	4.67	0.097	27	56	24	4	0.40	0.818
s49	2	2	30	53	21	0	1.60	0.450	24	62	27	1	1.23	0.541	30	44	12	3	7.58	0.023	31	53	26	1	0.60	0.741
s50	2	2	32	51	21	0	2.37	0.306	33	59	21	1	2.77	0.250	36	43	8	2	18.03	0.000	44	49	17	1	14.56	0.001
s51	X	1	61	0	42	1	3.50	0.061	54	58	0	2	0.14	0.705	50	0	37	2	1.94	0.163	0	50	59	2	0.74	0.389
s52	2	2	29	55	20	0	1.90	0.386	27	61	25	1	0.79	0.674	25	49	13	2	4.70	0.095	26	58	26	1	0.33	0.849
s53	2	2	27	50	26	1	0.11	0.948	33	52	28	1	1.16	0.560	34	36	17	2	9.23	0.010	33	50	27	1	1.56	0.458
s54	2	2	36	44	24	0	5.23	0.073	30	61	22	1	1.85	0.397	33	39	15	2	8.38	0.015	27	56	25	3	0.22	0.895
s55	X	1	58	0	46	0	1.38	0.239	52	61	0	1	0.72	0.397	50	0	37	2	1.94	0.163	0	54	54	3	0.00	1.000
s56	2	2	30	53	21	0	1.60	0.450	33	58	22	1	2.22	0.329	33	43	13	0	9.09	0.011	39	53	18	1	8.16	0.017
s57	2	2	24	56	24	0	0.62	0.735	30	59	24	1	0.86	0.651	26	46	17	0	1.92	0.383	29	56	25	1	0.33	0.849
s58	2	2	44	42	15	3	19.51	0.000	35	62	11	5	13.04	0.001	37	41	9	2	18.31	0.000	42	49	11	9	19.00	0.000
s59	2	2	58	37	9	0	54.83	0.000	63	42	8	1	60.98	0.000	32	46	11	0	10.01	0.007	32	60	18	1	4.47	0.107
s60	3	2	28	53	23	0	0.52	0.771	21	70	23	0	6.00	0.050	23	42	24	0	0.30	0.859	27	46	37	1	4.76	0.092
s61	2	2	27	53	24	0	0.21	0.900	29	59	26	0	0.30	0.861	30	41	18	0	3.79	0.151	29	55	26	1	0.16	0.921
